# P-1638. Mass SARS-CoV-2 Testing in 16 State Correctional Facilities During the Pandemic

**DOI:** 10.1093/ofid/ofaf695.1814

**Published:** 2026-01-11

**Authors:** Charlotte Kim, Byron Kennedy, Mary Lansing, Robert P Richeson, Albert I Ko, Margaret L Lind

**Affiliations:** Yale School of Medicine, West Hollywood, California; Connecticut Department of Correction, Wethersfield, Connecticut; Connecticut Department of Correction, Wethersfield, Connecticut; Connecticut Department of Correction, Wethersfield, Connecticut; Yale School of Public Health/Oswaldo Cruz Foundation/Brazilian Ministry of Health, New Haven, Connecticut; Boston University School of Public Health, Boston, Massachusetts

## Abstract

**Background:**

Within correctional facilities, mass screening has been shown to be a key strategy for SARS-CoV-2 infection capture and COVID-19 surveillance. However, no known studies have examined prolonged mass screening through the entire pandemic. This study assesses mass screening as performed by the Connecticut Department of Correction (CT DOC), which performed symptom-based screening, contact tracing, and biweekly mass screening throughout the pandemic, thus providing an opportunity to evaluate regular screening in respiratory viral control.

**Figure:**
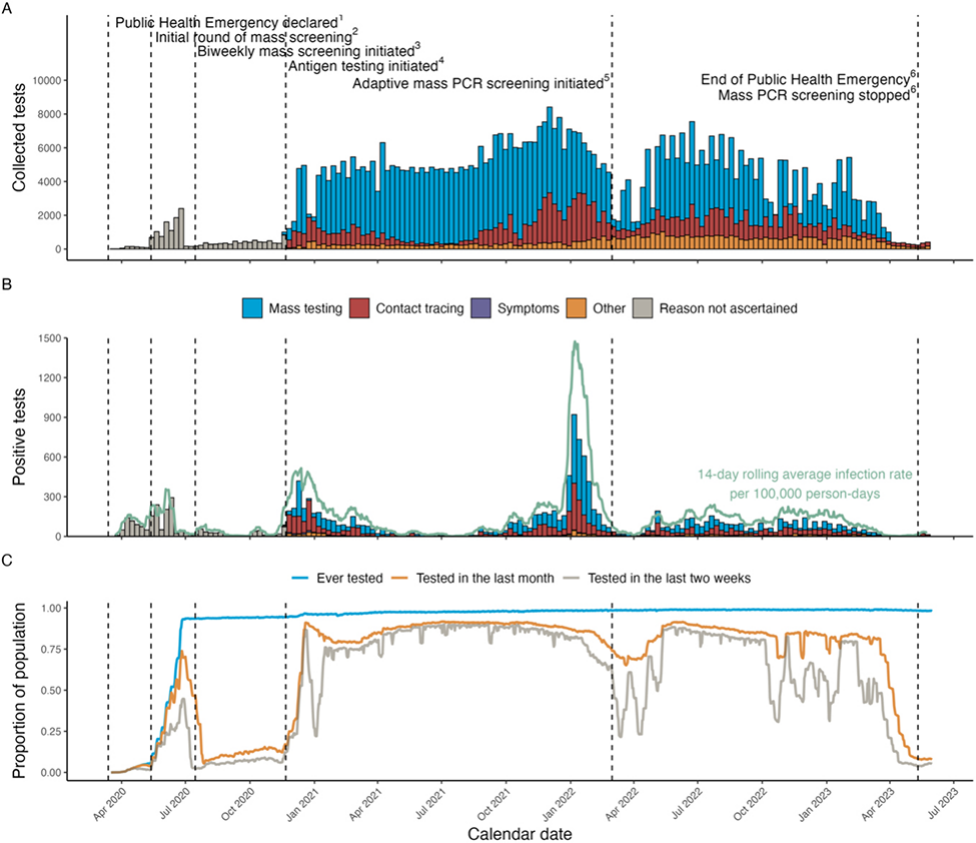
Weekly Numbers of Total and Positive SARS-CoV-2 Tests Among Incarcerated Persons, Stratified by Reason for Testing, and COVID-19 Incidence and Percent Tested in a State Correctional System from March 1, 2020 to May 31, 2023. Weekly recorded tests among incarcerated population by reason for testing (A), positive recorded tests among incarcerated population by reason for testing and 14-day rolling infection rate per 100,000 person-days (B), and percent of incarcerated population with a prior recorded test (C). The incarcerated population on any day was defined as the number of people with a recorded bed location. Infections were defined as a recorded positive test at least 90 days after the last recorded infection (if any) and the person-time in the 90 days following an infection were excluded from the incidence rate estimates. We defined a month as 30 days. Dates of events 1 to 6 are respectively, March 13, 2020, May 13, 2020, July 2020, November 21, 2020, March 2022, and May 11, 2023.

**Figure:**
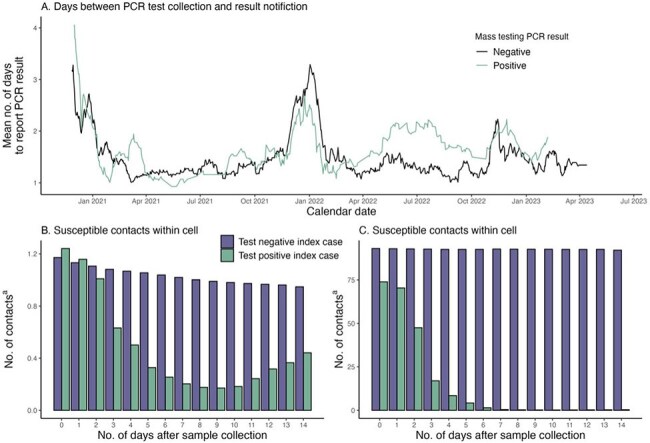
Time from PCR Sample Collection to Receiving Test Results During Study Period (A); Number of Exposed Contacts of Test-Positive and Test-Negative Contacts in Cell and Cellblock Settings (B) According to Days After Sample Collection in a State Correctional System. Number of days between SARS-CoV-2 RT-PCR test collection and test result notification for tests collected as part of mass-screening by test result (A), the average number of susceptible people within the same cell as someone who tested positive or negative for SARS-CoV-2 via mass screening for each day following test collection (B) and the average number of susceptible people within the same cellblock as someone who tested positive or negative for SARS-CoV-2 via mass screening for each day following test collection. Susceptible residents were defined as residents who had not tested positive for SARS-CoV-2 in the prior 90 days, including the day of exposure. Recorded location (bed) data was used to approximate resident location and, due to Connecticut Department of Correction stating that residents who tested positive were removed from the susceptible population for the duration of infection, we assumed infected residents were isolated from people of the same cellblock the day after positive test notification and the number of exposures following this time were equal to the within cell exposures(a).

**Methods:**

We analyzed SARS-CoV-2 surveillance data from the CT DOC between March 1, 2020 and May 31, 2023, which represented 29,719 recorded incarcerated people across five jails and eleven prisons. To ascertain the effect of mass screening on infection transmission, we used a linear regression to evaluate the change in the number of susceptible contacts after an index case tested positive by mass screening and was moved into isolation. To investigate infection capture, we compared SARS-CoV-2 prevalence among unvaccinated residents with community projection estimates from the Institute for Health Metrics and Evaluation (IHME) and COVIDESTIM, a Bayesian evidence synthesis model.

**Figure:**
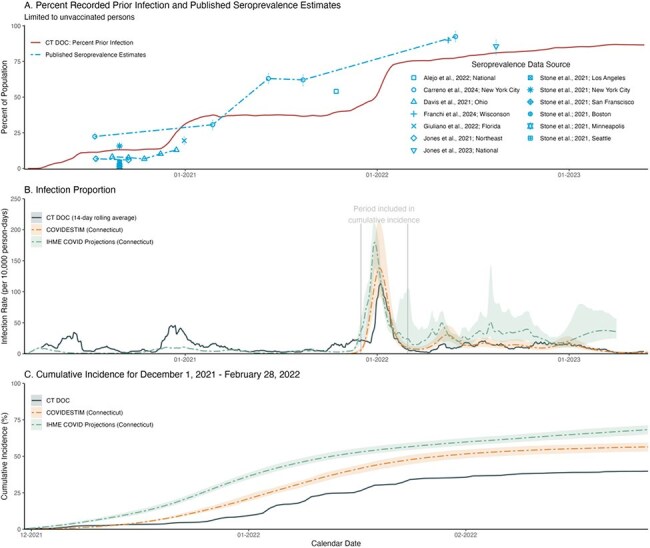
SARS-CoV-2 Recorded Infections Among People Incarcerated in a Connecticut Department of Correction Facility Compared to Community Estimates. Percent of unvaccinated people incarcerated since March 2020 with a recorded prior infection compared to published, unvaccinated seroprevalence estimates from the US (A); 14-day rolling infection proportion within incarcerated people and Connecticut-specific COVID-19 projection models (IHME COVID Projections and COVIDESTIM) (B); and cumulative incidence of SARS-CoV-2 among incarcerated people and projection models during the winter wave of 2021/2022 (December 2021 through February 2022) (C). We defined a SARS-CoV-2 infection as a recorded positive RT-PCR or rapid antigen test in the absence of an infection in the prior 90 days and excluded the 90 days following an infection from the prior infection and cumulative incidence analyses. COVIDESTIM provided weekly infection estimates which we converted to daily counts using Poisson regression with a natural cubic spline with knots at each data point. From both IHME and COVIDESTIM infection counts, we calculated daily infection proportions as infections divided by Connecticut's population. For cumulative infection rates, we estimated the probability of infection as one minus the probability of remaining uninfected each day (infections divided by state population minus previously infected individuals) and calculated cumulative risk as the product of these probabilities. Uncertainty from projection models was incorporated using posterior estimation, reporting means and 95% confidence intervals (2.5th-97.5th percentiles).

**Figure:**
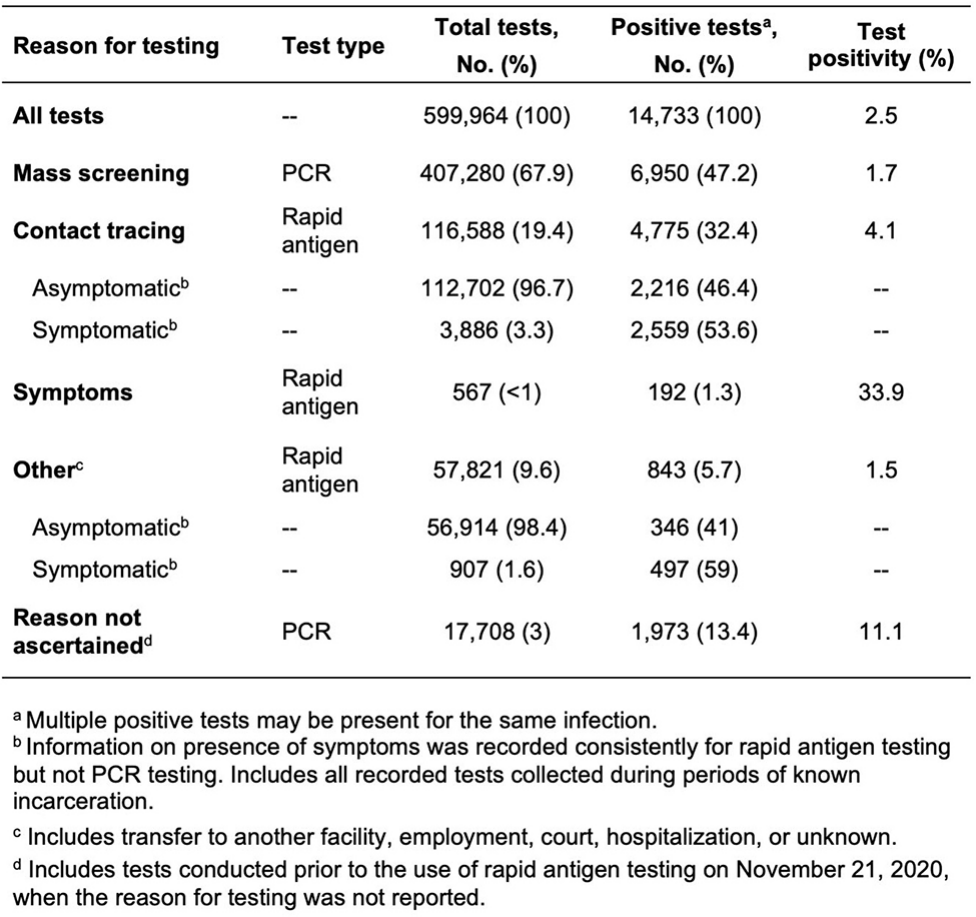
Reasons for Testing and Results of SARS-CoV-2 Tests Performed in a State Correctional System.

**Results:**

599,964 total SARS-CoV-2 tests were recorded with an overall positivity of 2.5%. Mass screening identified 47.2% of positive results, while contact tracing and symptom-based screening captured 32.4% and 1.3%, respectively. Isolation following testing positive by mass screening averted an estimated 877 exposure-days per test-positive person on average. However, during the winter 2021/2022 COVID-19 waves, cumulative incidence at the DOC was 28.5 (CI: 25.9–30.8) and 16.6 (CI: 14.1-20.0) percentage points lower than that of IHME and COVIDESTIM community projections, respectively. While the dense housing conditions of congregate settings, such as long-term care and correctional facilities, are expected to increase transmission risk, our results may suggest missed infections.

**Conclusion:**

Intensive mass screening is consistent with the timely identification and isolation of infectious cases in carceral facilities, but operational limitations may hinder control of respiratory pandemics in these high-risk settings.

**Disclosures:**

All Authors: No reported disclosures

